# Do cardiovascular disease patients return to pre-lockdown sedentary levels? A prospective cohort study

**DOI:** 10.1007/s12471-025-01966-z

**Published:** 2025-06-30

**Authors:** Janneke I. A. Vloet, Esmée A. Bakker, Bram M. A. van Bakel, Sophie H. Kroesen, Dick H. J. Thijssen, Thijs M. H. Eijsvogels

**Affiliations:** 1 Department of Medical BioSciences, Radboud University Medical Centre, Nijmegen, The Netherlands; 2 Department of Physical Education and Sports, Faculty of Sport Sciences, Sport and Health University Research Institute (iMUDS), University of Granada, Granada, Spain; 3 Department of Primary and Community Care, Radboud University Medical Centre, Nijmegen, The Netherlands; 4 Research Institute for Sports and Exercise Sciences, Liverpool John Moores University, Liverpool, UK

**Keywords:** Sedentary time, Physical inactivity, Cardiovascular Disease, COVID-19, Pandemics

## Abstract

**Background:**

The COVID-19 lockdown negatively impacted physical activity (PA) and sedentary behaviour (SB) levels of the Dutch patients with cardiovascular diseases (CVD), but little is known whether these levels returned to pre-pandemic levels. In this study, we evaluated changes in SB and moderate-to-vigorous PA (MVPA) in CVD patients before, during, and after the COVID-19 pandemic and investigated which factors contributed to not returning to pre-pandemic sedentary levels.

**Methods:**

1,028 Dutch CVD patients participated in this prospective cohort study, where we assessed SB and MVPA before (2018), during (2020), and after (2023) the COVID-19 pandemic using validated questionnaires. Linear mixed model analyses were used to investigate changes over time. Binary logistic regression analyses were performed to examine factors associated with not returning to pre-pandemic SB levels.

**Results:**

SB levels significantly increased from 7.8 h/day at pre-pandemic assessment to 8.7 h/day during lockdown and then significantly decreased to 8.5 h/day at the post-pandemic assessment, but did not return to pre-pandemic levels (*p* = 0.006). MVPA did not significantly change over time. Lower pre-pandemic SB levels, a larger increase in SB during lockdown, self-reported residual complaints after COVID-19, and diagnosis of arrhythmias at baseline were associated with not returning to pre-pandemic SB levels.

**Conclusion:**

Sedentary time in CVD patients did not return to pre-pandemic levels, 3 years following initial COVID-19 lockdown, while levels of MVPA did not change over time. These findings suggest that lifestyle interventions could be considered to reactivate CVD patients and lower their risk of disease progression and adverse health outcomes.

SB bij CVD-patiënten keerde niet terug naar het niveau van voor de pandemie, drie jaar na de eerste COVID-19 lockdown, terwijl MVPA onveranderd bleef. Deze bevindingen suggereren dat leefstijlinterventies overwogen kunnen worden om CVD-patiënten opnieuw te activeren en hun risico op ziekteprogressie en nadelige gezondheidseffecten te verlagen.

**Supplementary Information:**

The online version of this article (10.1007/s12471-025-01966-z) contains supplementary material, which is available to authorized users.

## What’s new?

- Longitudinal assessment revealed that sedentary behaviour levels did not return to pre-pandemic levels (8.5 *versus* 7.8 h/day) in patients with cardiovascular diseases.
- Lower baseline sedentary behaviour levels, larger increases in sedentary time during the lockdown, the presence of residual complaints after COVID-19, and the presence of cardiac arrhythmias were associated with the inability to return to pre-pandemic sedentary behaviour levels.

## Background

Sedentary behaviour (SB) is independently associated with a higher risk of mortality and morbidity, including cardiovascular diseases (CVD) [1, 2], whereas the magnitude of these risks increases at lower habitual physical activity (PA) levels [3]. Hence, current guidelines recommend that adults and older individuals perform at least 150 min/week of moderate-to-vigorous-intensity PA (MVPA), combined with limiting SB as much as possible [4]. A physically active lifestyle is of great importance for CVD patients, as it attenuates disease progression, reduces symptoms, and lowers the risk of morbidity and mortality [5–8].

The COVID-19 pandemic and associated lockdown restrictions had a negative impact on the lifestyle of individuals globally [9], with differential effects on sub-populations. We and others have previously shown the detrimental effects of the COVID-19 lockdown measures on lifestyle characteristics in CVD patients [10–13]. A net reduction of habitual PA was found, primarily driven by increases in SB (+1.1 h/day) [10]. The gradual lifting of lockdown measures did not result in normalisation of SB characteristics in CVD patients [11], raising the question of its long-term impact and whether SB returned to pre-pandemic levels in the years after the pandemic.

**Fig. 1:**
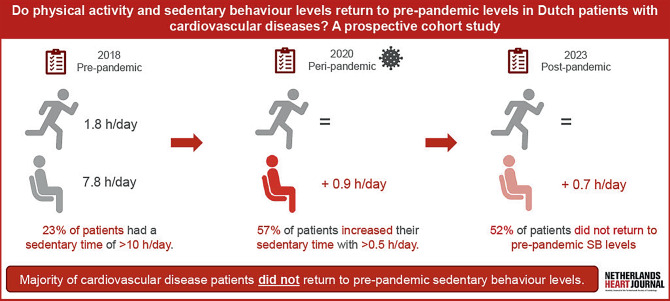
Infographic

We prospectively evaluated changes in SB before, during, and 3 years after initiation of the COVID-19 lockdown in patients with CVD (Fig. 1). We also explored which patient and disease characteristics were associated with failure to return to pre-pandemic SB levels, as such information could be used to identify patients at risk of sedentary lifestyle. Changes in time spent MVPA were included as a secondary outcome.

## Methods

We invited CVD patients who had participated in a previous multicentre study (*n* = 1,565) via email to participate in this longitudinal cohort study assessing changes in self-reported SB and MVPA before, during, and after the COVID-19 lockdown [10, 14]. Participants were recruited at four Dutch hospitals (i.e., Radboudumc, Rijnstate Hospital, Jeroen Bosch Hospital, and Isala Clinic) in collaboration with the Dutch Heart Foundation. Inclusion criteria were CVD diagnosis and/or referral to cardiac rehabilitation between 2015 and 2018. The study conforms to the ethical guidelines of the Declaration of Helsinki and was approved by the local medical ethics committee (ref. 2020-6414). All participants provided informed consent. SB and MVPA were assessed at three timepoints: 1) pre-lockdown (April-October 2018), 2) peri-lockdown (April 2020) and 3) post-lockdown (September-November 2023), using online, validated questionnaires. During the pre- and post-lockdown periods, no societal restrictions applied. Peri-lockdown, restrictions involved closure of public facilities, bars, restaurants, schools, and sports clubs, and the recommendation to stay at home as much as possible, including working from home. When outside, social distancing was recommended, with the instruction to meet maximum of one other person a day.

### Assessment of SB and MVPA

SB was assessed using the Sedentary Behaviour Questionnaire (SBQ) [15], determining sedentary time (ST) in nine distinct everyday life activities (i.e., watching television, using a tablet, computer or game console, eating and drinking, listening to music, talking on the phone, reading, doing arts and crafts, deskwork, and transportation by car, bus or train). Total daily ST was calculated by multiplying weekday estimates by five and weekend day estimates by two, then dividing the sum by seven.

Time spent at MVPA was assessed using the Short Questionnaire to Assess Health-Enhancing Physical Activity (SQUASH), which is validated for this purpose [16]. The Metabolic Equivalent of Task (MET) concept was used to determine the appropriate intensity of activities. A MET-score ≥ 3.0 is considered to correspond to moderate intensity according to the World Health Organisation 2020 guidelines on physical activity and sedentary behaviour [17], so all activities with a MET-score ≥ 3.0 [18] were included. Participants reporting a single behaviour (SB or MVPA) for > 18 h/day were deemed unreliable and excluded from further analyses.

### Other covariates

Self-reported information on age, sex, BMI, employment status, and cardiovascular health status were collected via our online questionnaire. In addition, the post-lockdown assessment included questions about experienced COVID-19 infections (number and severity of infection(s)) and presence of residual complaints after infection (e.g., shortness of breath, fatigue, chest pain, dry cough, headache, nausea, muscle soreness) or diagnosis of long COVID (persistence of residual complaints for more than 3 months) by a general practitioner or medical specialist.

### Statistical analysis

Data were reported as number (%) for categorical variables and as mean ± SD (normally distributed) or median [interquartile range] (non-normally distributed) for continuous variables. Data were checked for normality through visual inspection. Differences over time for SB and MVPA were examined using linear mixed-model analyses, adjusted for age, sex and BMI at baseline, with sensitivity analyses for SB on weekdays and weekend days seperately. An increase in SB between 2018 and 2023 > 0.5 h/day was defined as not returned to pre-pandemic SB levels [19]. Univariable binary logistic regression analyses were performed to identify variables associated with not returning to pre-pandemic SB levels. Variables with *p* < 0.2 in the univariable analysis were included in the multivariable binary logistic regression model. All other statistical analyses were two-sided, with a significance level of α = 0.05. Analyses were performed using R version 4.3.0 and RStudio version 2023.12.1 + 402, with the packages ‘lme4’ [20], ‘mlogit’ [21], ‘ggplot2’ [22] and ‘tidyverse’ [23].

## Results

A total of 1,028 patients participated in the current study (response rate 65.7%) resulting in 984 (95.7%) complete cases for the MVPA analyses and 864 (84.0%) complete cases for the SB analyses (Electronic Supplementary Material Fig. S1). At baseline, participants had a median age of 65 [59, 71] years; were predominantly male (72.5%); were mostly retired (63.0%); and had a median BMI of 26.4 kg/m^2^ [24.0, 28.8] (Tab 1). Participants who completed the post-lockdown questionnaire did not significantly differ from those lost to follow-up in age, sex, BMI, employment status, CVD-subtypes, and SB and MVPA at baseline (Electronic Supplementary Material Table S1).

**Table 1:** Patient characteristics of the analytical cohort upon inclusion

Patient characteristics	All patients *n* = 1,028	Returned to pre-pandemic SB levels *n* = 440	No return to pre-pandemic SB levels *n* = 473	*p*-value
Age (years)	65 [59, 71]	65 [59, 61]	65 [59, 61]	0.76
Sex (male, %)	745 (72.5%)	339 (77.0%)	321 (67.9%)	0.003
BMI (kg/m^2^)	26.4 [24.0, 28.8]	26.6 [24.3, 28.9]	26.3 [23.9, 28.7]	0.995
Employment status (retired, %)	644 (63.0%)	260 (59.1%)	312 (66.0%)	0.03
*CVD-diagnosis*^*a*^				
Myocardial infarction, *n* (%)	504 (49.2%)	218 (49.5%)	232 (49.0%)	0.90
Angina pectoris, *n* (%)	216 (21.1%)	96 (21.8%)	93 (19.7%)	0.46
Cardiac arrythmias, *n* (%)	209 (20.4%)	77 (17.5%)	106 (22.4%)	0.08
Heart valve disease, *n* (%)	118 (11.5%)	51 (11.6%)	53 (11.2%)	0.93
Heart failure, *n* (%)	112 (10.9%)	47 (10.7%	50 (10.6%)	> 0.99
Other^b^, *n* (%)	180 (17.6%)	71 (16.1%)	90 (19.0%)	0.30
*COVID-19 characteristics*				
COVID-19 infection, *n* (yes, %)	528 (52.9%)	237 (53.9%)	236 (49.9%)	0.19
COVID-19 vaccination, *n* (≥ 1, %)	967 (96.7%)	411 (93.4%)	457 (96.6%)	0.08
Self-reported residual complaints after COVID-19, *n* (%)	84 (16.0%)	30 (6.8%)	48 (10.1%)	0.03
Long COVID, *n* (%)	12 (14.5%)	4 (1.0%)	6 (1.3%)	> 0.99

*BMI* Body Mass Index, *CVD* Cardiovascular Disease,* SB* Sedentary Behaviour

^a^CVD subtype was based on main diagnosis at the time of the initial study period (2018)

^b^Other was defined as congenital heart disease, stroke and peripheral artery disease

### Changes in SB and MVPA

**Fig. 2:**
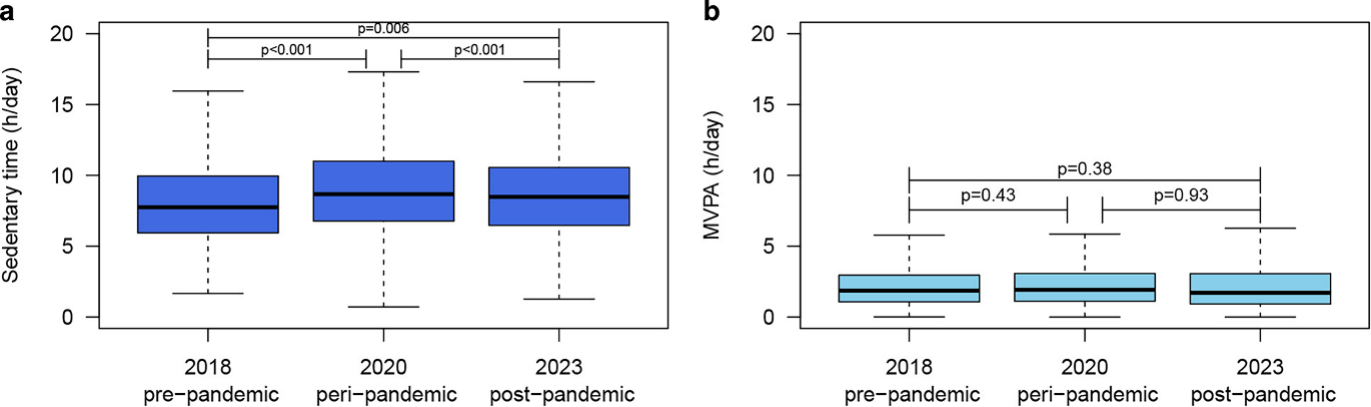
Sedentary time (**a**) and MVPA (**b**) in hours per day of the cohort participating in the current study (*n* = 1028). Data is shown of pre- (2018), peri- (2020), and post-lockdown (2023) timepoints. The boxplots visualise median values with IQR and total variation across the population. *MVPA *moderate-to-vigorous physical activity

**Fig. 3:**
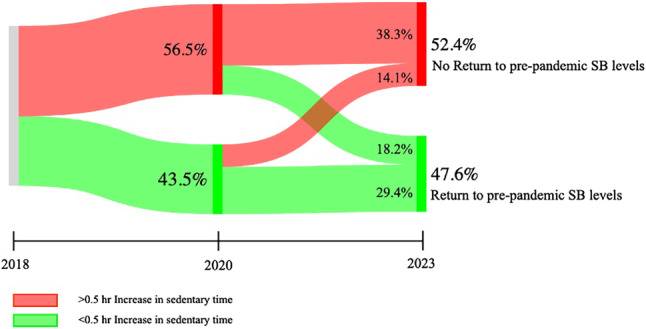
Sankey plot of the flow of participants over time. Numbers represent percentages of participants of complete cases with SB data (*n* = 859). No return to pre-pandemic TB levels was defined as an increase of > 0.5 h/day over the total study period (2018 versus 2023). Percentages in red indicate an increase of > 0.5 hr/day when compared to the assessment in 2018. Percentages in green indicate a decrease in SB or an increase < 0.5 hr/day when compared to assessment in 2018. *SB *Sedentary Behaviour

SB increased from 7.8 [5.8, 9.8] h/day in 2018 to 8.7 [6.6, 10.8] h/day in 2020, and subsequently showed a significant decrease to 8.5 [6.4, 10.5] h/day in 2023 (Fig. 2). Nevertheless, SB remained higher in 2023 compared with 2018 (*p* < 0.01). We found that 52.4% of patients in our cohort showed an increase of > 0.5 hr/day in SB and therefore did not return to pre-pandemic SB levels (2018 *versus* 2023). A total of 38% of patients initially increased SB during lockdown (2018 *versus *2020) and maintained this behaviour post-pandemic (2020 *versus *2023), whereas an additional 14% of patients only reported increased SB levels during the post-lockdown questionnaire (Fig. 3). Patients not returning to pre-pandemic SB levels were more often male and more often reported residual complaints after a COVID-19. All other patient characteristics did not differ between groups (Tab. 1). Sensitivity analyses showed the same pattern in SB for both weekdays and weekend days separately as the pattern reported for total SB. Moreover, descriptive statistics show structurally higher sedentary time during weekdays than during weekend days at all three timepoints (Electronic Supplementary Material Table S2). In contrast, MVPA levels did not change over time (2018: 1.8 [0.9, 2.8], 2020: 1.9 [1.0, 2.9], 2023: 1.7 [0.6, 2.8] h/day).

Based on the results of the univariable binary logistic regression analyses, retirement at baseline, higher SB levels at baseline, female sex, larger increase in SB levels during the pandemic, diagnosis of cardiac arrhythmias at baseline, and the presence of self-reported residual complaints after COVID-19 were included in the multivariable model Electronic Supplementary Material Fig. S2). The multivariable model revealed that higher pre-pandemic SB levels (OR: 0.79 [0.73, 0.87], *p* < 0.001), larger increase in SB pre- *versus* peri-pandemic (OR: 1.29 [1.20, 1.38], *p* < 0.001), the presence of residual complaints after COVID-19 (OR: 2.43 [1.32, 4.46], *p* = 0.004) and the diagnosis of cardiac arrhythmia at baseline (OR: 2.01 [1.15, 3.51], *p* = 0.01) were independently and significantly associated with the inability to return to pre-pandemic SB levels (Electronic Supplementary Material Fig. S3). Retirement and female sex were not associated with this inability.

## Discussion

We prospectively evaluated changes in SB and MVPA before, during, and 3 years after the COVID-19 pandemic in CVD patients. We found no significant changes in MVPA across timepoints (2018: 1.9 [0.9, 2.8] h/day versus 2020: 1.9 [1.0, 2.9] h/day versus 2023: 1.7 [0.6, 2.8] h/day), suggesting that CVD patients maintained their MVPA patterns. However, SB substantially increased during the pandemic, and despite a small, but significant decrease 3 years thereafter, SB levels did not return to pre-pandemic levels in 52.4% of CVD patients (2018: 7.8 [5.8, 9.8] h/day versus 2020: 8.7 [6.6, 10.8] h/day versus 2023: 8.5 [6.4, 10.5] h/day). The inability to return to pre-pandemic SB levels was associated with lower baseline SB levels, larger increase during lockdown, presence of residual complaints after COVID-19 infection, and the presence of cardiac arrhythmias at baseline. These findings provide important information on the long-term effects of the COVID-19 pandemic on habitual physical (in)activity levels of CVD patients and may be used to optimise secondary prevention strategies in CVD patients.

To our knowledge, this is the first study to prospectively investigate the effects of the COVID-19 pandemic on SB years after initial measures were taken. Our approach allowed us to compare intra-individual levels of SB before, during, and after the COVID-19 lockdown. Previous studies reported an acute increase in SB during the lockdown [10, 12, 24], as individuals were instructed to work from home, PA opportunities were restricted, and social interactions reduced [12]. Nevertheless, research on whether SB and PA levels return to pre-pandemic levels years after the pandemic, and potential explanations for this behaviour change, is lacking. Our post-pandemic findings are alarming. Although SB levels were slightly decreased post- versus peri-pandemic, time spent sedentary remained 10% higher compared to pre-pandemic levels. Previous studies reported an annual, age-related increase of SB ranges of 0 to 1% [25, 26], suggesting that the post-pandemic SB levels are likely attributable to the COVID-19 lockdown and not to aging alone. We observed that 52.4% of our population showed an increase of > 30 min in SB at post- versus pre-pandemic assessment. At group level, MVPA levels did not change over time, despite increases in odd jobs during lockdown restrictions [10, 11]. However, individual MVPA patterns may have varied. The increases in SB may induce deleterious health outcomes, such as increased cardiometabolic risk diseases and all-cause mortality [27]. Van Bakel et al. showed that patients’ lifestyles were impeded by lack of social contact, fear of a SARS-CoV‑2 infection and limited PA possibilities [11]. These determinants may have contributed to the development of changes in sedentary and physical activity habits.

Several factors were associated with the inability to return to pre-pandemic SB levels. The binary logistic regression model had an explained variance of 87%, indicating good fit. Those with higher baseline SB levels were more likely to return to or remain at their pre-pandemic SB levels; since these levels were already high (e.g. > 10 h/day 23% of the study population at baseline), further increases would be unlikely. These patients would remain at the same SB levels, and therefore were defined as ‘returned to pre-pandemic SB levels’. Moreover, larger increases in SB during the lockdown were associated with a higher risk for the inability to return to pre-pandemic SB levels. Large increases in SB during the lockdown may be due to anxiety for infection and recommendations to work from home, limiting active transportation and PA during working hours, however, this was not validated in the questionnaire. Independent of these reasons, upon lifting the lockdown measures, CVD patients may have adapted their lifestyle to these SB patterns, and substantially changing them seems difficult [11]. To our knowledge, the finding that the presence of a cardiac arrhythmia diagnosis at baseline was an associated factor with not returning to pre-pandemic SB levels was not earlier reported in literature. Further research on this topic is warranted. Finally, we also found that the presence of residual complaints after COVID-19 was associated with a lack of return to pre-pandemic SB levels. Patients with a (self-reported) diagnosis of long COVID experience limited exercise capacity and, in many cases post-exertional malaise. These symptoms promote a sedentary lifestyle due to discomfort during PA. At the very least, these observations highlight the difficulty to target a sedentary lifestyle following substantial changes in SB as observed during the lockdown.

Physical inactivity increases the risk for cardiometabolic diseases, obesity and all-cause mortality, especially in this CVD population [14, 28]. CVD patients are characterised by a more sedentary lifestyle, while even PA at light or moderate intensity can provide significant health benefits and prevents further decline in cardiovascular health. This highlights that our observation of the excessive post-pandemic SB levels is clinically relevant, and that a focus should be on reengaging this population in PA and decrease SB. The sensitivity analyses showed no differences in patterns of changes over time when separated for week and weekend days. However, sedentary times were significantly higher at each individual timepoint, suggesting that CVD patients are overall more physically active during weekend days. More insight on physical activity patterns and individual habits could increase knowledge on the motivation of patients on changing their SB or PA patterns. Therefore, more research is needed to optimize lifestyle programmes with evidence-based guidelines. Targeted lifestyle programmes could offer a solution to improve daily life activity patterns [29, 30].

Strengths of this study are the large study population and longitudinal nature of the study design. 66% of participants included in the previous study responded to the invitation. Participants did not differ from those lost to follow-up in MVPA and SB levels and other patient characteristics during pre-pandemic assessment. Limitations to this study include the use of questionnaires, which rely on self-reported MVPA and SB levels, making questionnaires prone recall bias and PA overestimation and SB underestimation. However, an important advantage is that this study involves within-subject analyses, whilst the validated SQUASH and SBQ show good reproducibility [16, 31]. Therefore, valid representation of change over time is presented. Moreover, relatively many SB datapoints are missing due to incomplete questionnaires. A potential explanation for this is that the SBQ was obtained after the SQUASH. The nature and context of PA levels are dependent on individual habits and patterns, such as longitudinal (cardiovascular) health status, hospitalisation(s), and psychological and social factors concerning the COVID-19 pandemic. Whilst we were unable to correct for these factors, we were able to add retirement status as a factor to the binary logistic regression model. This additional analysis showed that retirement status was not associated with not returning to pre-pandemic SB levels. Finally, the observational design precludes causal inference. We therefore cannot draw any causal conclusions from the results.

## Conclusion

Dutch CVD patients did not return to pre-pandemic SB levels three years after initiation of the COVID-19 lockdown. Failure to revert was associated with lower baseline SB, larger initial increases, residual symptoms post-COVID-19, and baseline cardiac arrhythmias. These findings underscore that lifting restrictions alone does not restore pre-lockdown activity, and that sedentary time remains 10% higher than before the pandemic. Given the associated health risks and unchanged MVPA, the preventive cardiology community should prioritise interventions to reduce SB and promote PA among CVD patients.

## Supplementary Information

**Supplementary Fig. S1 **Flowchart of study inclusion in 2023. *SQUASH* Short Questionnaire to Assess Health-enhancing physical activity, *SBQ* Sedentary Behaviour Questionnaire.

**Supplementary Table S1 **Patient Characteristics of the patients included in this follow-up study and patients who were lost to follow-up. Results include median [IQR] or *n* (%).

**Supplementary Fig. S2 **Results of the univariable binary logistic regression model. Variables were selected based on literature and relevance and were tested individually. Odds ratios > 1 represent an increased chance of not returning to pre-pandemic SB levels while odds ratios < 1 represent an increased chance of returning to pre-pandemic lockdown SB levels. Variables with *p* < 0.2 were selected for the multivariable binary logistic regression model. MVPA Moderate-to-Vigorous Physical Activity, CVD Cardiovascular Disease.

**Supplementary Fig. S3 **Forest plot of the multivariable binary logistic regression model. Including the odds ratios [95%CI] of the variables included in the final model which were associated with the odds of not returning to pre-pandemic ST levels. Odds ratios > 1 represent an increased chance of not returning to pre-pandemic SB levels while odds ratios < 1 represent an increased chance of returning to pre-pandemic lockdown SB levels.

**Supplementary Table S2** Sedentary times of all participants included in the current follow-up study (*n* = 1,028). Numbers representing sedentary times during week and weekend days in hours. Differences were between groups were tested using paired t‑tests. Results include mean ± SD.
