# Supplementary material for: Do cardiovascular disease patients return to pre-lockdown sedentary levels? A prospective cohort study
Source: Neth Heart J. 2025 Jun 30;33(7-8):232–8. doi: 10.1007/s12471-025-01966-z (PMC12274157; doi:10.1007/s12471-025-01966-z)
Supplement: Supplementary file 1 — Supplementary Fig. S1 Flowchart of study inclusion in 2023. SQUASH Short Questionnaire to Assess Health-enhancing physical activity, SBQ Sedentary Behaviour Questionnaire. [file 12471_2025_1966_MOESM1_ESM.docx]

Respondents

follow-up April 2020

*n=1,565*

Included patients 2023

*n=1,028 (66%)*

Physical activity analysis

*n=984 (96%)*

Sedentary behaviour analyses

*n=864 (84%)*

Lost to follow-up

- Medical reasons: 8
- Passed away: 21
- No interest: 16
- Reason unknown: 492

Respondents

Initial questionnaire 2018

*n=2,584*
