# Supplementary material for: Do cardiovascular disease patients return to pre-lockdown sedentary levels? A prospective cohort study
Source: Neth Heart J. 2025 Jun 30;33(7-8):232–8. doi: 10.1007/s12471-025-01966-z (PMC12274157; doi:10.1007/s12471-025-01966-z)
Supplement: Supplementary file 2 — Supplementary Table S1 Patient Characteristics of the patients included in this follow-up study and patients who were lost to follow-up. Results include median [IQR] or n (%). [file 12471_2025_1966_MOESM2_ESM.docx]

**Supplementary Table 2** Patient characteristics of the patients included in this follow-up study and patients who were lost to follow-up. Results include median [IQR] or n (%).

| Patient characteristics | Completed questionnaire in 2023  *n*=1,028 | Lost to follow-up  *n*=537 | *p*-value |
| --- | --- | --- | --- |
| Age (years) | 65 [59, 71] | 65 [58, 73] | 0.44 |
| Sex (male, %) | 745 (72.5%) | 400 (74.5%) | 0.46 |
| BMI (kg/m^2^) | 26.4 [24.0, 28.8] | 26.5 [24.2, 28.8] | 0.25 |
| Employment status (retired, %) | 644 (63.0%) | 307 (57.2%) | 0.07 |
| CVD-diagnosis  Heart failure, n (%)  Myocardial infarction, n (%)  Heart valve disease, n (%)  Cardiac arrythmias, n (%)  Angina pectoris, n (%)  Other^b^, n (%) | 112 (10.9%)  504 (49.2%)  118 (11.5%)  209 (20.4%)  216 (21.1%)  180 (17.6%) | 68 (12.7%)  283 (52.7%)  62 (11.5%)  106 (19.7%)  123 (22.9%)  86 (16.0%) | 0.27  0.20  0.46  0.43  0.33  0.33 |
| MVPA (h/day)  SB (h/day) | 1.86 [0.92, 2.80]  7.75 [5.74, 9.76] | 1.86 [0.93, 2.79]  7.45 [5.42, 9.49] | 0.98  0.25 |
| BMI Body Mass Index; CVD Cardiovascular Disease  ^a^CVD subtype was based on main diagnosis at the time of the initial study period (2018).  ^b^Other was defined as congenital heart disease, stroke and peripheral artery disease. | | | |
