# Supplementary figures and images for: Do cardiovascular disease patients return to pre-lockdown sedentary levels? A prospective cohort study
Source: Neth Heart J. 2025 Jun 30;33(7-8):232–8. doi: 10.1007/s12471-025-01966-z (PMC12274157; doi:10.1007/s12471-025-01966-z)

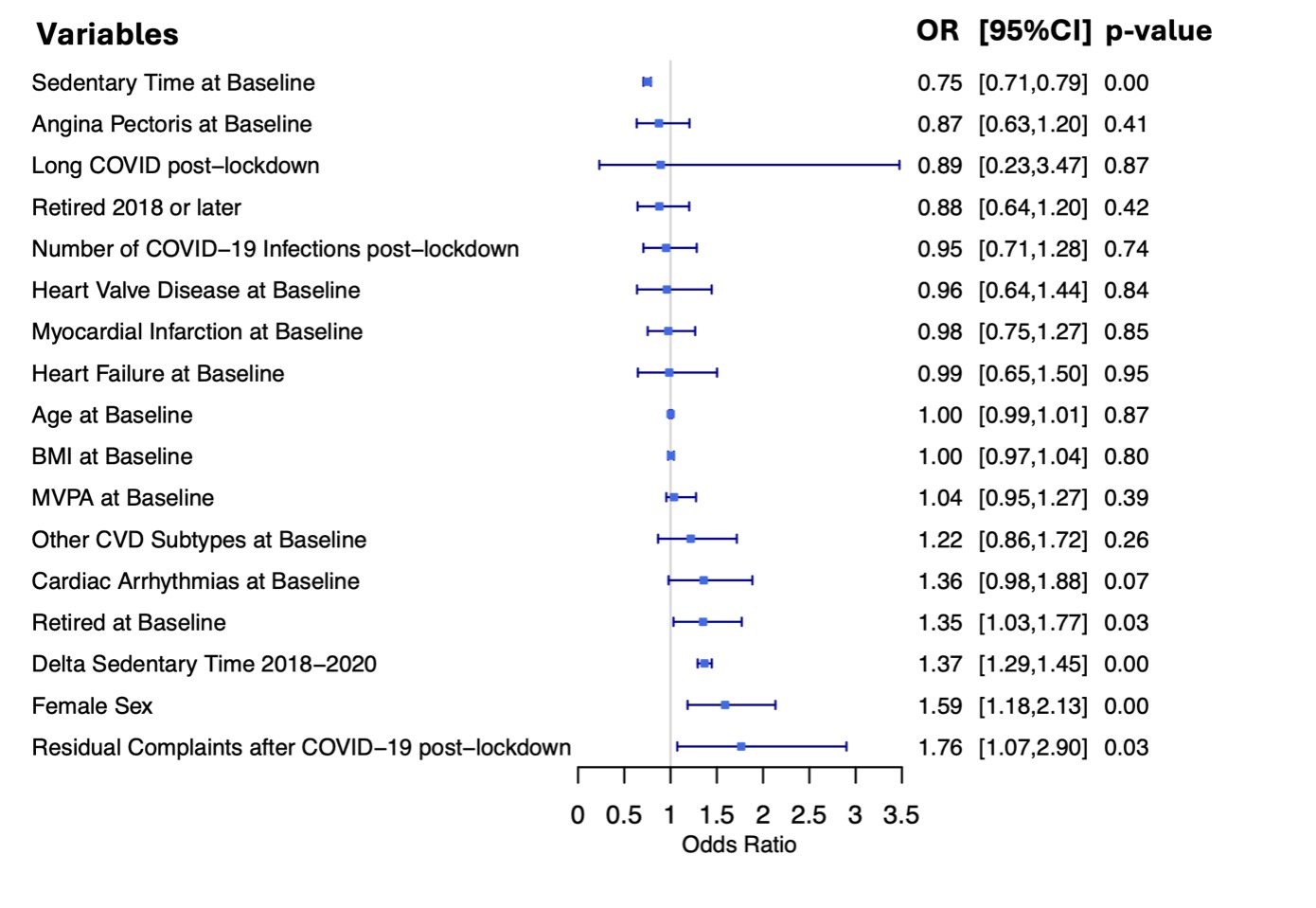

Supplement: Supplementary file 3 — Supplementary Fig. S2 Results of the univariable binary logistic regression model. Variables were selected based on literature and relevance and were tested individually. Odds ratios > 1 represent an increased chance of not returning to pre-pandemic SB levels while odds ratios < 1 represent an increased chance of returning to pre-pandemic lockdown SB levels. Variables with p < 0.2 were selected for the multivariable binary logistic regression model. MVPA Moderate-to-Vigorous Physical Activity, CVD Cardiovascular Disease. [file 12471_2025_1966_MOESM3_ESM.jpg]

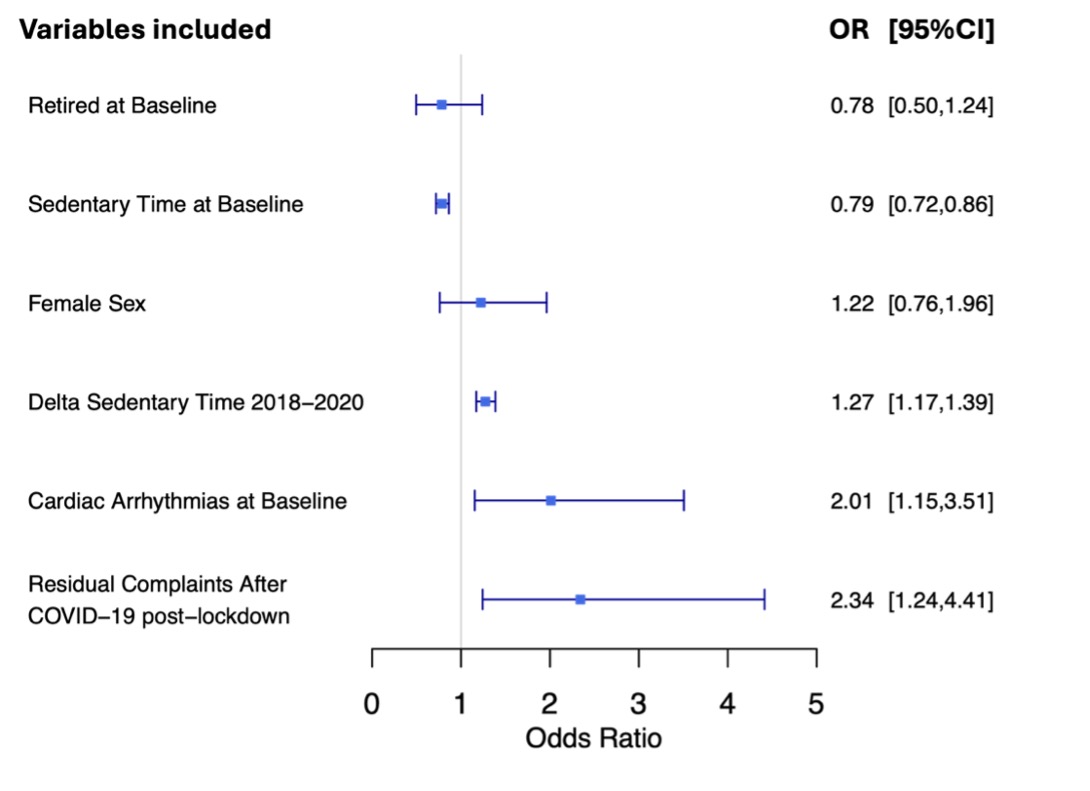

Supplement: Supplementary file 4 — Supplementary Fig. S3 Forest plot of the multivariable binary logistic regression model. Including the odds ratios [95%CI] of the variables included in the final model which were associated with the odds of not returning to pre-pandemic ST levels. Odds ratios > 1 represent an increased chance of not returning to pre-pandemic SB levels while odds ratios < 1 represent an increased chance of returning to pre-pandemic lockdown SB levels. [file 12471_2025_1966_MOESM4_ESM.jpg]
