# Supplementary material for: Do cardiovascular disease patients return to pre-lockdown sedentary levels? A prospective cohort study
Source: Neth Heart J. 2025 Jun 30;33(7-8):232–8. doi: 10.1007/s12471-025-01966-z (PMC12274157; doi:10.1007/s12471-025-01966-z)
Supplement: Supplementary file 5 — Supplementary Table S2 Sedentary times of all participants included in the current follow-up study (n = 1,028). Numbers representing sedentary times during week and weekend days in hours. Differences were between groups were tested using paired t‑tests. Results include mean ± SD. [file 12471_2025_1966_MOESM5_ESM.docx]

| Timepoint | Sedetary time weekdays | Sedentary time weekenddays | *p*-value |
| --- | --- | --- | --- |
| 2018 | 7.9 ± 3.1 | 7.6 ± 2.9 | <0.001 |
| 2020 | 8.9 ± 3.3 | 8.5 ± 3.1 | <0.001 |
| 2023 | 8.6 ± 3.1 | 8.3 ± 3.2 | <0.001 |

**Supplementary Table 2** Sedentary times of all participants included in the current follow-up study (*n*=1,028). Numbers representing sedentary times during week and weekend days in hours. Differences were between groups were tested using paired t-tests. Results include mean ± SD.
